# Epidemiological investigation of swine Japanese encephalitis virus based on RT-RAA detection method

**DOI:** 10.1038/s41598-022-13604-4

**Published:** 2022-06-07

**Authors:** Mincai Nie, Yuancheng Zhou, Fengqin Li, Huidan Deng, Mengxi Zhao, Yao Huang, Chaoyuan Jiang, Xiangang Sun, Zhiwen Xu, Ling Zhu

**Affiliations:** 1 College of Veterinary Medicine, Sichuan Agricultural University, Chengdu, 611130 China; 2 College of Veterinary Medicine Sichuan Key Laboratory of Animal Epidemic Disease and Human Health, Sichuan Agricultural University, Chengdu, 611130 China; 3 Livestock and Poultry Biological Products Key Laboratory of Sichuan Province, Sichuan Animal Science Academy, Chengdu, 610066 China; 4 Xichang University, Chengdu, 615000 Sichuan China

**Keywords:** Biological techniques, Molecular biology

## Abstract

JEV is one of the zoonotic pathogens that cause serious diseases in humans. JEV infection can cause abortion, mummified foetus and stillbirth in sows, orchitis and semen quality decline in boars, causing huge economic losses to pig industry. In order to investigate the epidemiology of JEV in pigs in Sichuan province, a rapid and efficient fluorescent Reverse transcription recombinase-aided amplification (RT-RAA) detection method was established. Aborted fetuses and testicular swollen boar samples were detected by RT-RAA in pigs in the mountain areas around Sichuan Basin, and the detection rate of JEV was 6.49%. The positive samples were identified as JEV GI strain and GIIIstrain by sequencing analysis. We analyzed the whole gene sequence of a positive sample for the GI virus. The Envelope Protein (E protein) phylogenetic tree analysis was far related to the Chinese vaccine strain SA14-14-2, and was most closely related to the JEV GI strains SH17M-07 and SD0810 isolated from China. The results showed that we established an efficient, accurate and sensitive method for clinical detection of JEV, and JEV GI strains were prevalent in Sichuan area. It provides reference for the prevention and control of JEV in Sichuan.

## Introduction

Japanese Encephalitis virus (JEV) is a zoonotic virus of the genus flavivirus, damagecentral nervous system disease in both humans and animals. JEV is an endemic disease around the world, including Russia, China, Japan, India, Australia and Southeast Asia, with about 68,000 reported cases each year, and half of which occurred in China^1^. In human infections, most cases showed mild clinical symptoms, such as headache, fever and lethargy. However, severe neurological disorders, such as paralysis, memory deficits and seizures can occure sometimes, it can kill up to 40 percent of patients with severe illness^2,3^. Swine are the main host of JEV in livestock and poultry. Pigs infected with JEV usually suffer from reproductive disorders and^4^. Which brings great economic losses to pig industry. The JEV is mainly transmitted by mosquitoes. The climate in Sichaun is warm and humid, and large areas of rice cultivation nurture a large number of mosquitoes, forming a continuous viral cycle between mosquitoes and pigs, resulting in the widespread epidemic of JEV in Sichuan^5^. At present, there is no specific drug for the treatment of JEV infection, theprevention and control of JEV can only be carried out through vaccination^6^. Vaccinating pigs not only provides specific protection to pigs, but also breaks the cycle of transmission, thereby reducing the threat to human health. However, after the outbreak of African swine fever (ASF), the pig industry has rebounded, a large number of breeding sows and boars has been put into production, and the incidence of reproductive disorders has increased, which is of great significance for the formulation of JEV prevention and control strategies.

Many methods have been used in the epidemiological investigation of JEV. Including virus isolation, ELISA, RT-PCR, RT-QPCR, Droplet Digital PCR (ddPCR), etc. Virus isolation is time-consuming and labor-intensive, taking more than a week to complete. In serological investigation using ELISA, it is difficult to analyze the results due to the cross-reaction between flaviviruses^7^. Serum Neutralization Test (SNT) is the reference method for serological detection of JEV, but there may be cross-reaction and it is necessary to detect other flaviviruses of the same genus as JEV to obtain correct results^8^. RT-PCR, RT-qPCR, ddPCR and other polymerase chain reaction based detection methods also need 2–3 h to complete^9–11^. We have establishes a real-time RT-RAA method with simple operation, strong specificity and high sensitivity. RT-RAA is an emerging nucleic acid detection method. Its principle is to add recombinase and single chain binding protein and other elements into the amplification system, so that the nucleic acid amplification can be rapidly amplified at 39 °C, and the results can be obtained within 10–30 min. We established the RT-RAA method for rapid detection of JEV, and used this method to conduct an epidemiological investigation of JEV in pig farms in Sichuan. The epidemiological data were analyzed in order to provide reference for the prevention and control of JEV in pig farms of Sichuan province in China.

## Materials and methods

### Establishment of RT-RAA detection method

#### Source of virus and sample

JEV/SC/2016-1 strain was provided by Sichuan Zoology Biotechnology Co., LTD^12^. Clinical samples were collected from 185 aborted fetuses and testicular with swelling in mountain areas around Sichuan Basin. The Sichuan provincial laboratory management committee (LicenceNo: SYXK (chuan) 2019–187) approval has been received. The “Guidelines for Experimental procedure” of the Ministry of Science and Technology (Beijing, China) were followed.

#### Primer design and synthesis

The sequence between E960-1100 in the reference sequence was analyzed, and the homology between the JEV strains was 79.8–100% (Supplementary Fig. 1). Choose to design primers between E960-E1100. A pair of RT-PCR primers, three RT-RAA forward primers, three RT-RAA reverse primers and one RT-RAA probe were designed according to the E protein sequence of JEV included in GenBank (Supplementary Table 1). The primers and probes were synthesized by Shanghai Sangong Bioengineering Technology Service Co., LTD.

#### RNA extraction

Cofitt®Total RNA Reagent (Cofitt Life Sciences, Kowloon, HK, China) was used to extract RNA from virus samples and clinical samples according to the kit instructions, and RNA was stored at − 80 °C.

#### Establishment of standard

Extract the RNA of JEV-SC-1, use PrimeScript™ RT Master Mix (Perfect Real Time) (Takara, Kusatsu, Shiga, Japan) to reverse transcribe the cDNA, and then use RT-PCR primers to amplify.. The product gel was recovered and ligated with pMD 19-T simple Vector and transformed into *E.coli* DH5α receptor cells. After culture, the plasmid was identified by PCR and sent to Shanghai Sangong Bioengineering Technology Service Co., LTD for sequencing. Bacteria with correct sequencing results were expanded and cultured, and plasmids were extracted using a plasmid extraction kit. The plasmids were linearized and digested with mMESSAGE mMACHINE™ T7 Transcription Kit (Thermo Fisher Scientific, Waltham, MA, USA) for in vitro transcription to obtain standard plasmid transcription^13^. This product is purified and aliquoted and stored at − 80 °C.

#### RT-RAA system and primer screening

Fluorescent RT-RAA use a fluorescent RT-RAA nucleic acid amplification kit (Jiangsu Qitian Gene Biotechnology, Wuxi, Jiangsu, China), and the RT-RAA reaction system refers to the kit instruction (Supplementary Table 2). Immediately after the system was configured, it was transferred to a fluorescence quantitative PCR instrument preheated at 39 °C. Denaturation temperature, annealing temperature and elongation temperature were all set at 39 °C, each cycle was 1 min, and 30 cycles were set. Nuclease—free water was used as negative control.

Three forward primers and three reverse primers were combined into 9 pairs of primers, 1–9 were F1/R1, F1/R2, F1/R3, F2/R1, F2/R2, F2/R3, F3/R1, F3/R2, and F3/R3 in sequence. The JEV cDNA was amplified by RT-PCR using the combination primers (Supplementary Table 3). The amplified bands were recycled by gel and then connected to pUCm-T Vector. It was transformed into *E. coli* DH5α for identification of expanded culture, and then sent to Shanghai Sangong Bioengineering Technology Service Co., LTD for sequencing to verify whether the sequencing results were JEV sequences. RT-RAA was performed on the primer combinations identified as positive by RT-PCR, and the primers were screened by fluorescence value and onset time.

#### Specificity and sensitivity tests of RT-RAA

The RNA extracted from JEV, swine fever virus (CSFV), blue ear virus (PRRSV), geta virus (GETV), epidemic diarrhea virus (PEDV) and infectious gastroenteritis virus (TGEV) were detected by the established RT-RAA fluorescence detection method to evaluate the specificity of the established method.

The recombinant plasmid transcript was diluted 10 times gradient by ddH_2_O, and the diluted plasmid transcript was detected by RT-RAA to evaluate the sensitivity of the established method.

### Epidemiological investigation of JEV in Sichuan

#### Clinical sample testing

From September 2020 to September 2021, 185 samples of aborted fetuses and testicular swollen boars were collected from mountain area around Sichuan Basin, Sichuan Province. Fluorescent RT-RAA and Diagnostic Kit for JEV RNA (RT-PCR Fluorescence Probing) (Guangzhou VIPOTION) Biotechnology, Guangzhou, Guangdong, China) were used to detect samples simultaneously. RT-qPCR system and reaction procedure refer to the kit instruction (Supplementary Table 4).

#### Whole gene sequencing of positive sample

The JEV whole gene primers were segmented according to the whole gene sequence of JEV registered in GenBank (Supplementary Table 5). The positive sample was amplified by PCR using the whole gene primers, and the amplification products were gel recovered and linked with pMD 19-T simple vector and transformed into *E. coli* DH5α sensitive cells. After culture, the plasmid was identified by PCR and sent to Shanghai Sangong Bioengineering Technology Service Co., LTD for sequencing.

#### Phylogenetic analysis

DNAMan software (DNASTAR Inc, Madison, WI, USA) was used to splice the sequence and then sequence analysis was performed (Supplementary Table 6). Based on best fit evolutionary model (TN93+G), the Maximum likelihood tree was constructed by using the Nearest-Neighbor-Interchange (NNI) and 1000 bootstraps.

## Results

### Establishment of RT-RAA method

#### Primer screening results

**Figure 1:**
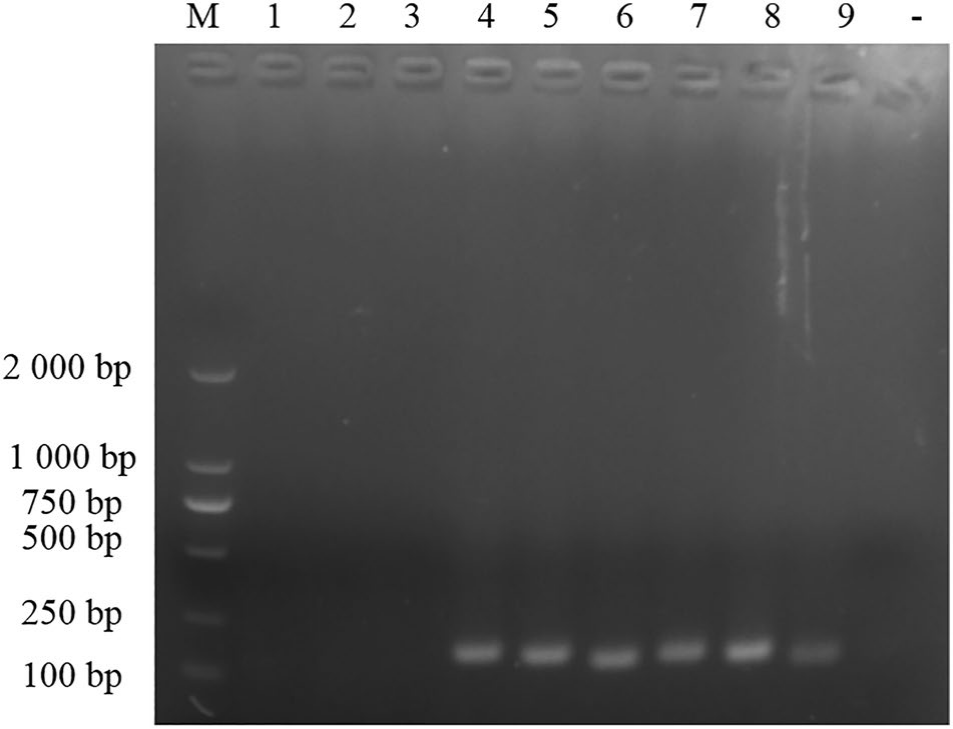
Primer screening results of RT-PCR: M: DL2000 Marker, 1–9 : pairs of JEV primers, -: negative control.

**Figure 2:**
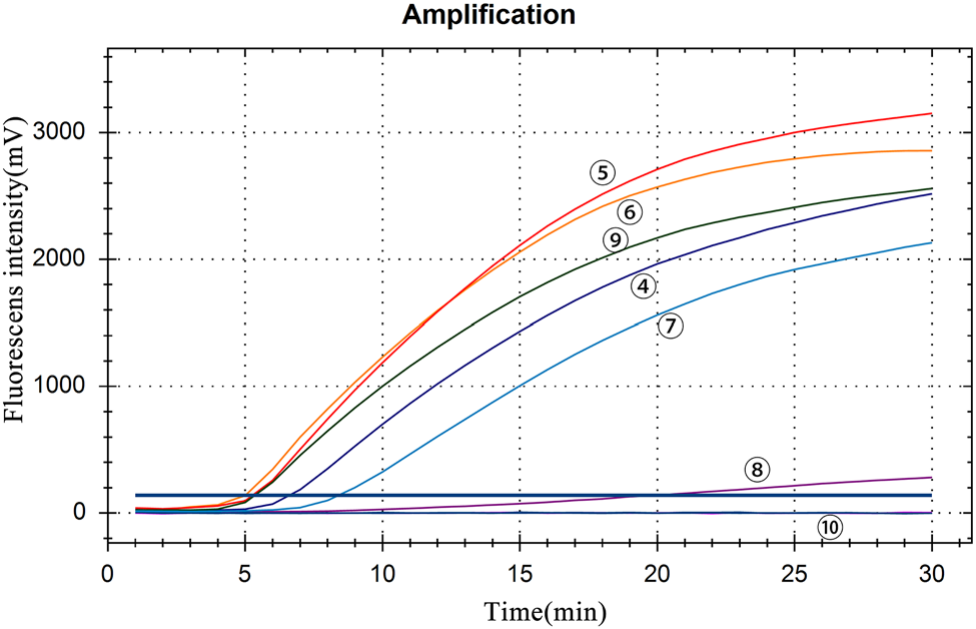
Primer screening results of RT-RAA. ④–⑨: pairs of primers, ⑩: negative control. The fluorescens intensity of the curve: ⑤: 3153 mV, ⑥: 2858 mV, ⑨: 2559 mV, ④: 2517 mV, ⑦: 2131 mV, ⑧:280 mV.

In the RT-PCR test of 9 pairs of primers, only combinations 4–9 could amplify JEV-specific bands (Fig. 1), which were identified as JEV sequences by recycled sequencing. The combination of primer 4–9 was used for RT-RAA test, and the combination of JEV-RAA-F2 and JEV-RAA-R2 caused the shortest peak time and the highest reaction fluorescence peak (Fig. 2). Therefore, JEV-RAA-F2 and JEV-RAA-R2 were selected as RT-RAA primers for the experiment.

#### Specificity and sensitivity of RT-RAA

**Figure 3:**
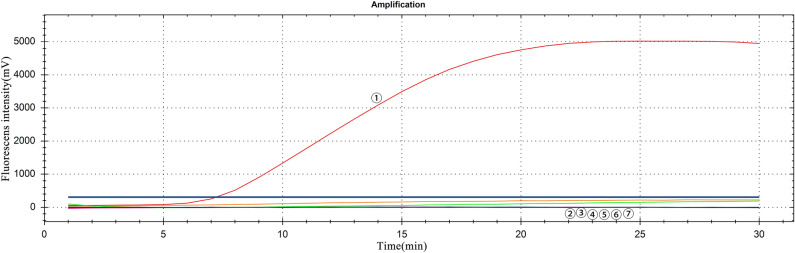
Specific test results: ①: GETV RNA, ②–⑥: JEV, CSFV, PRRSV, PEDV, TGEV RNA, ⑦: negative control.

In the specificity test, only JEV RNA could generate fluorescence curves, and none of the other viral RNA generated a fluorescence curve (Fig. 3), indicating that the established RT-RAA had good specificity.

**Figure 4:**
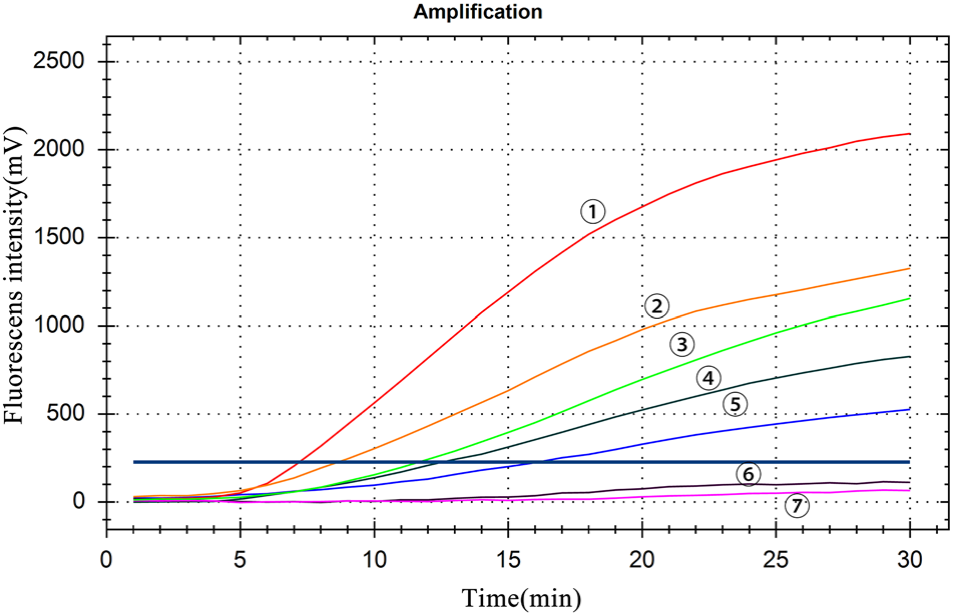
Sensitivity test results: ①–⑥: plasmid transcript concentration are 5.5 × 10^4^–5.5 × 10^–1^ copies/μL, ⑦: negative control.

The sensitivity of RT-RAA was assessed using a recombinant plasmid transcript concentration of 5.5 × 10^4^–5.5 × 10^–1^ copies/μL, and the lowest detection concentration of RT-RAA was 5.5 copies/μL (Fig. 4).

### Epidemiological investigation of JEV in Sichuan

#### Clinical sample testing

Our results showed 11 samples were positive by RT-RAA and RT-qPCR detection. 1 sample was positive by RT-RAA detection butnegative by RT-qPCR detection. The positive coincidence rate of RT-RAA and RT-qPCR was 100%. The positive rate was 6.49% (Table 1). Among them, 11 positive samples were of GIstrain, and only one positive sample was of GIII strain.

**Table 1:** Clinical samples test results.

Sample type	Area	Quantity	Organ analyzed	Positive number	Positive rates	Strain	Season
Aborted fetuses	Liangshan Prefecture	87	Placenta	4	4.6%	GI	Summer
Aborted fetuses	Ganzi Prefecture	15	Placenta	1	6.7%	GIII	Spring
Aborted fetuses	Panzhihua City	14	Placenta	1	7.1%	GI	Spring
Aborted fetuses	Aba prefecture	32	Placenta /Umbilical cord	3	9.4%	GI	Autumn
Aborted fetuses	Dazhou	21	Placenta	2	9.5%	GI	Summer
Testicular enlargement boars	Liangshan Prefecture	9	Testicular fluid	1	11.1%	GI	Spring
Testicular enlargement boars	Dazhou	7	Testicular fluid	0	0	–	–

#### Positive sample sequencing

Among the 12 positive samples, we obtained one JEV whole gene sequence and five JEV E gene sequences by sequencing analysis. The full gene sequence was named JEV-SC-2020-1, and the full length of the genome was 10,964 bp. The E gene sequences were named SCAB-202009, SCGZ-202002, SCLS-202002, SCLS-202005, SCPZH-202003. The gene sequences were uploaded to GenBank with accession numbers OK423757, ON416988, ON416989, ON416990, ON416991, ON416992.

#### Phylogenetic analysis

**Figure 5:**
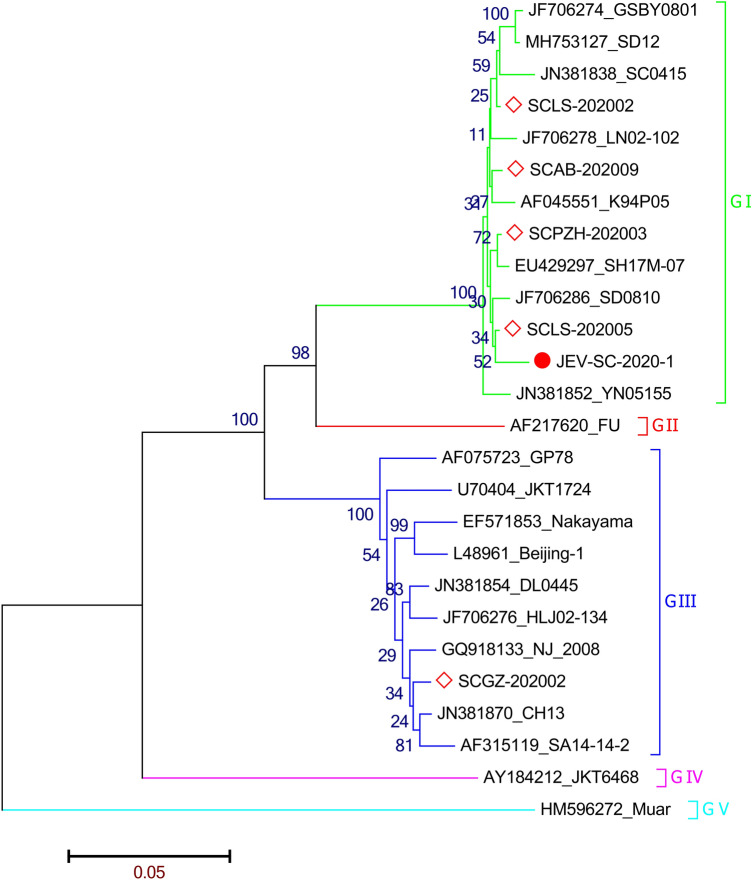
Evolutionary tree analysis of E protein.

The phylogenetic tree analysis of E protein gene of JEV-SC-2020-1 showed that JEV-SC-2020-1 belonged to GI strain and was most closely related to SH17M-07 strain isolated from Shanghai in 2007 and SD0810 strain isolated from Shandong in 2009. It has a distant relationship with Chinese vaccine strain SA14-14-2 (Fig. 5).

The JEV E protein is a key region of the epitope, an important component that determines the virulence and antigenicity of JEV. The E proteingene homology between JEV-SC-2020-1 and the reference strain sequence was 98.4–76.6%, among which the homology with SD0810 was the hinghest, and the homology with Muar strain of GV strain was the lowest. The homology with Chinese vaccine strain SA14-14-2 was 87.3% (Supplementary Fig. 2). The E protein amino acid homology of JEV-SC-2020-1 with other reference sequences was 100%-91.6%, and the homology with GSBY0801, SD12, LN02-102, YN05155 and SD0810 was the highest. The homology of E protein with the Muar strain of GV strain was the lowest (Supplementary Fig. 3).

JEV-SC-2020-1 and the attenuated SA-14-14-2 strain share 14 amino acid mutations in the E protein, and share 8 amino acid mutations with the virulent vaccine strain Bejing-1 strain. In the key epitope region of E protein (E337-345, E377-382, E397-403)^14^, JEV-SC-2020-1 is completely consistent with SA-14-14-2, and Bejing-1 is only in E397 a mutation (Supplementary Fig. 4).

## Discussion

RAA (Recomninase Aided Amplification) is a new thermostatic Amplification technology proposed in recent years. RAA has been widely used in pathogen detection due to its fast detection speed, strong specificity and high sensitivity^15^. In this study, we constructed a rapid detection RT-RAA method using JEV E protein sequnce to detection the virus. It can detect multiple genotypes of JEV, providing a wider range of JEV detection The RT-RAA detection method established in this study directly added reverse transcriptase into the reaction system, which is simpler and more convenient than the conventional PCR detection using cDNA to configure the PCR system after reverse transcription. The combination of recombinant enzyme, single chain binding protein and DNA polymerase in RAA system enables rapid amplification of nucleic acid at constant temperature, and the addition of fluorescent probe enables real-time monitoring of amplification reactions. For the JEV RT-RAA fluorescence detection method in this study, the amplification curve could be seen after reaction at 39 °C for 10 min, and the result could be judged after 30 min. However, the RT-LAMP and RT-qPCR take about 1 h for detection JEV, and the RT-PCR takes longer time than those two^16,17^. The detection limit for JEV plasmid transcripts was 5.5 copies/μL. It is similar to JEV RT-LAMP and TaqMan RT-qPCR^9,16^, slightly higher than SYBR Green I RT-qPCR, and 100 times higher than RT-PCR^10^. The fluorescence detection method of JEV RT-RAA established in this study is rapid, sensitive and specific, and can be used for clinical diagnosis.

JEV is one of the main arboviruses in my country and one of the main pathogens that cause reproductive failure in pigs. To investigate the prevalence of JEV in Sichuan, we performed JEV detection on 185 collected clinical samples using the established JEV RT-RAA. In the detection of 185 clinical samples, we detected 12 JEV positive cases, with a positive rate of 6.49%. JEV still accounts for a high proportion of abortion cases. The reason is the unique geographical location of mountain area around Sichuan Basin. Due to the lack of water, a large number of reservoirs have been built to store water, and these reservoirs are good shelters for mosquitoes^18^, which increase the risk of JEV transmission. In addition, under the influence of ASF epidemic, farmers paid less attention to JEV and the vaccination rate declined. Hence the prevalence of JEV increased in pigs^19^.

Among the 12 positive samples, there were 11 JEV GIstrains and 1 JEV GIII strain.strain The GIstrain was first identified in the 1980s, originated from Southeast Asia and then rapidly spread to the entire Asian continent^20^. After 2000, GIstrain replaced G III strain and become the pandemic strain in China, Japan, South Korea, Vietnam and Thailand^21–25^. After thatstrain, the number of clinical cases of JEV decreased, because the less virulent characteristic of the GI strain. However, study also proved that GIand GIII viruses have similar infection rates in asymptomatic infected patients, indicating that GIand GIII were equally virulent, and this conclusion was also verified in mice experiments^26,27^.

By analyzing the amino acid mutation sites of E protein of JEV-SC-2020-1 strain, it was found that JEV-SC-2020-1 was completely consistent with SA14-14-2 strain in the key epitope region of E protein, and had a mutation with Bejing-1. It is speculated that vaccines of SA14-14-2 and Bejing-1 strains can provide protection for JEV-SC-2020-1, but the protective efficacy of SA14-14-2 may be higher than that of Bejing-1. In previous studies, it has been believed that GIII strain vaccine can provide good immune protection effect against species genotype virus. However, recent studies have found that JEV vaccine inoculated with GIII strain has reduced neutralization efficacy against GIstrain JEV^28^. It has also been found that some individuals vaccinated with GIII JEV vaccine have a reduced ability to neutralize antibodies against different GIstrain strains^29^. This has raised concerns about whether differences in the presence of antigens between strains of different genotypes could affect the effectiveness of the vaccine. This has also accelerated the development of a new vaccine for JEV.

## Supplementary Information

Supplementary Information 1.

Supplementary Information 2.

## Data Availability

The datasets supporting the conclusions of this article are included within the article and its tables and figures. Additional data may be available from the corresponding author upon reasonable request.
