# Supplementary material for: Epidemiological investigation of swine Japanese encephalitis virus based on RT-RAA detection method
Source: Sci Rep. 2022 Jun 7;12:9392. doi: 10.1038/s41598-022-13604-4 (PMC9172605; doi:10.1038/s41598-022-13604-4)
Supplement: Supplementary file 1 — Supplementary Information 1. [file 41598_2022_13604_MOESM1_ESM.docx]

**Epidemiological investigation of swine Japanese encephalitis virus based on RT-RAA detection method**

Mincai Nie^a,1^, Yuancheng Zhou^c,1^, Fengqin Li^a,d^, Huidan Deng^a^, Mengxi Zhao^a^, Yao Huang^a^, Chaoyuan Jiang^a^, Xiangang Sun^a^, Zhiwen Xu^a,b^, Ling Zhu^a,b*^

Supplementary Table 1: Primer and probe sequences

| Name | Sequence |
| --- | --- |
| JEV-RAA-F1 | ATCCTYCTGCTGTTGGTCGCTCCGGCTTACAG |
| JEV-RAA-R1 | CTTCGATGTTAATCATGCGGACGTCCAATGTT |
| JEV-RAA-F2 | CTCCGGCTTACAGTTTYAATTGTCTGGGAATG |
| JEV-RAA-R2 | ATCATRCGGACRTCYAATGTTGGTT TGTCG |
| JEV-RAA-F3 | TGTCTGGGAATGGGCAATCGTGACTTCATAG |
| JEV-RAA-R3 | TGGTTTGTCGTTTGCCATGATTRTCAAGCAGC |
| JEV-PCR-F4 | GATTCAACGAAAGCCACACGAT |
| JEV-PCR-R4 | TGTAAACTTTGCCGCCTGGGA |
| JEV-RAA-P | AAGGAGCYAGTGGAGCYACTTGGGTGGACY/i6FAMdT//idSp/G/iBHQdT/  GTAGAAGGAGA |

Supplementary Table 2: RT-RAA reaction system

| Component | Concentration(μM) | Volume(μL) |
| --- | --- | --- |
| Tris-HCl Buffer | pH7.6 | 25 |
| Forward primer | 10 | 2.1 |
| Reverse primer | 10 | 2.1 |
| Probe | 10 | 0.6 |
| ddH_2_O and RNase inhibitor |  | 2 |
| template |  | 3 |
| total |  | 50 |

Supplementary Table 3: RT-PCR system and reaction procedure

| RT-PCR | | | RT-PCR | | |
| --- | --- | --- | --- | --- | --- |
| Component | Concentration(μM) | Volume(μL) | Temperature | Time | Cycle |
| 2×TaqPCR master Mix | - | 5.0 μL | 95℃ | 4min | 1 |
| cDNA | - | 1.0 μL | 95℃ | 30s | 35 |
| Forward primer | 10 | 0.5 μL | 56℃ | 10s |  |
| Reverse primer | 10 | 0.5 μL | 72℃ | 30s |  |
| ddH_2_O | - | 3.0 μL | 72℃ | 5min | 1 |

Supplementary Table 4: RT-qPCR system and reaction procedure

| RT-qPCR system | | RT-qPCR reaction procedure | | |
| --- | --- | --- | --- | --- |
| Component | Volume(μL) | Temperature | Time | Cycle |
| JEV reaction liquid | 20μL | 42℃ | 20min | 1 |
| Enzyme liquid | 1μL | 95℃ | 10min | 1 |
| RNA | 4μL | 94℃ | 15s | 40 |
|  |  | 55℃ | 30s |  |

Supplementary Table 5: JEV whole gene primer

| Name | Sequence | Location | Length |
| --- | --- | --- | --- |
| F1 | AACTTCTTGGCTTAGTATCGTTG | 6-1223 | 1238 |
| R1 | TACACACGTAACTGCTGTCGG |  |  |
| F2 | ATCAACATTGAAGCTAGCCAAC | 1102-2536 | 1455 |
| R2 | ATCTACCCAAGCCTCTACGTC |  |  |
| F3 | GGGTAAAGCCTTCTCAACAAC | 2175-3530 | 1378 |
| R3 | CTGAAAAGGGTCAATCATTTCGC |  |  |
| F4 | AGCTGTTCTCTACCGCCTT | 3409-4881 | 1492 |
| R4 | GTTCCACCACGATCACTTGC |  |  |
| F5 | GGGATTCTCGGCACTTACCA | 4681-6124 | 1464 |
| R5 | CTTTTCCCTCTCTGGCCCGTA |  |  |
| F6 | TCCTTGGAAACCCATCCCC | 5924-7185 | 1282 |
| R6 | CAGCCAAGAAAGACAAGACCA |  |  |
| F7 | CCTGAAGCACCTAATCACCTC | 7065-8431 | 1385 |
| R7 | TGCCCAGGTTGACATCCTC |  |  |
| F8 | CGCCTTCCCCTGTCTCGAAA | 8287-9724 | 1458 |
| R8 | TCTGACCTTCGACATTGCGTT |  |  |
| F9 | GTGACCTACGCTCTCAACACA | 9487-10689 | 1223 |
| R9 | ATTGCATCTTAGACGAGGCTT |  |  |

Supplementary Table 6: Details of the reference sequence

| Name | Year | Host | Country | Genotype | Accession number |
| --- | --- | --- | --- | --- | --- |
| GSBY0801 | 2008 | Culex tritaeniorhynchus | China | GⅠ | JF706274 |
| SD12 | 2015 | Swine | China | GⅠ | MH753127 |
| SC0415 | 2004 | Culex tritaeniorhynchus | China | GⅠ | JN381838 |
| K94P05 | 1999 | --- | Korea | GⅠ | AF045551 |
| LN02-102 | 2002 | Culex modestus | China | GⅠ | JF706278 |
| SH17M-07 | 2007 | --- | China | GⅠ | EU429297 |
| SD0810 | 2009 | Culex tritaeniorhynchus | China | GⅠ | JF706286 |
| YN05155 | 2005 | Culex tritaeniorhynchus | China | GⅠ | JN381852 |
| FU | 1995 | Human | Australia | GⅡ | AF217620 |
| Nakayama | 1935 | Human | Japan | GⅢ | EF571853 |
| Beijing-1 | 1949 | Human | China | GⅢ | L48961 |
| JKT1724 | 1979 | Mosquito | USA | GⅢ | U70404 |
| DL0445 | 2004 | Armigeres subalbatus and Mansonia uniformis | China | GⅢ | JN381854 |
| HLJ02-134 | 2002 | Culicoides sp. | China | GⅢ | JF706276 |
| NJ 2008 | 2009 | --- | China | GⅢ | GQ918133 |
| GP78 | 1978 | Human | India | GⅢ | AF075723 |
| CH13 | 1957 | CSF | China | GⅢ | JN381870 |
| SA14-14-2 | 1989 | --- | China | GⅢ | AF315119 |
| JKT6468 | 1981 | Mosquito | Indonesia | GⅣ | AY184212 |
| Muar | 1952 | Human | Malaysia | GⅤ | HM596272 |


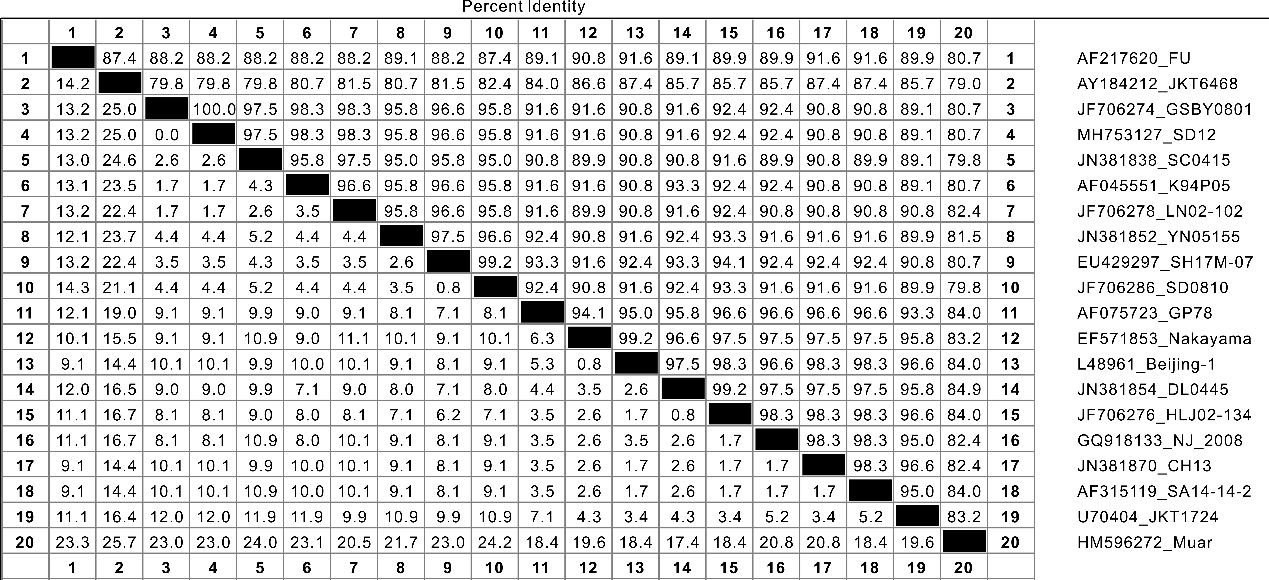


Supplementary Fig.1: JEV E960-E1100 Sequence Homology Analysis


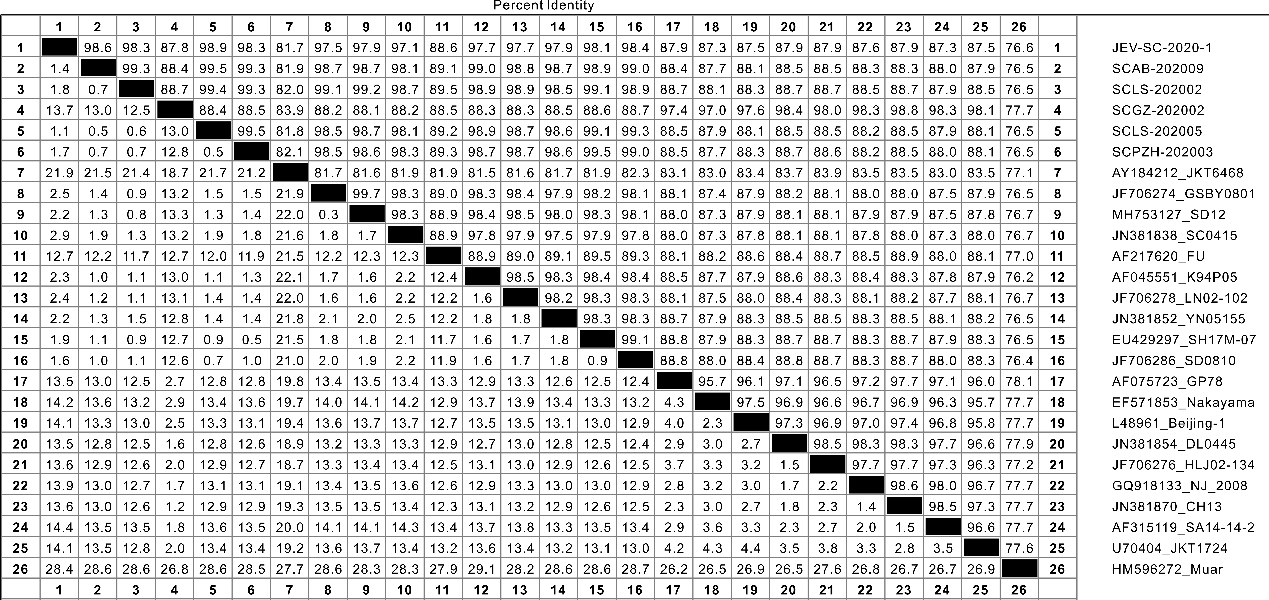


Supplementary Fig.2: E protein sequence homology


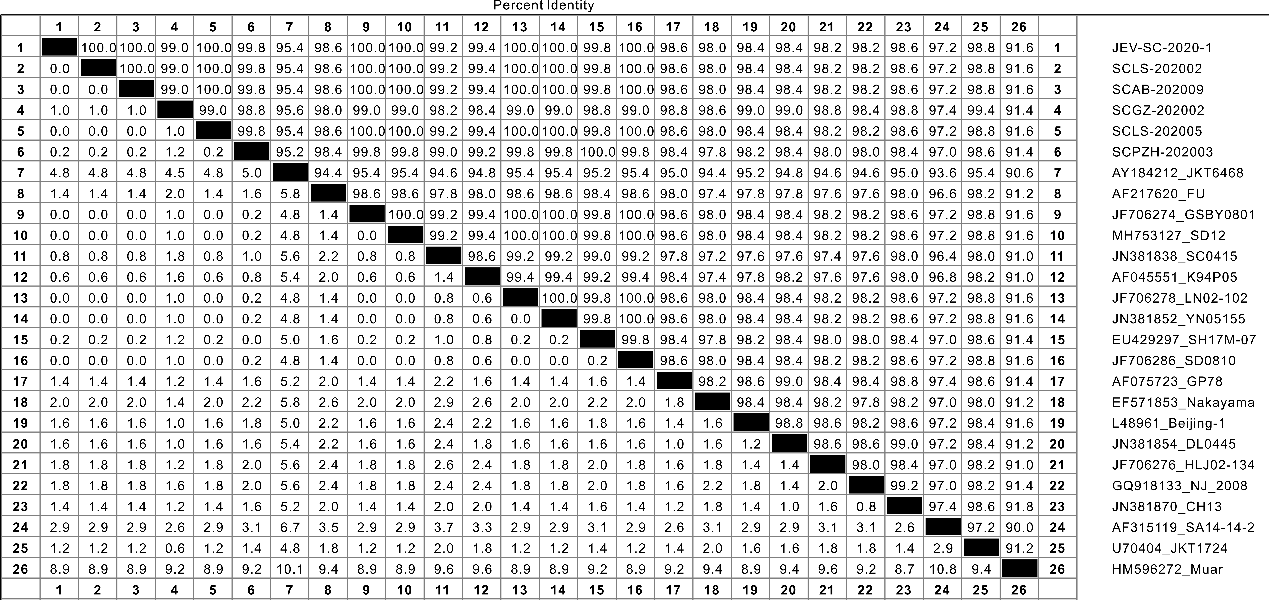


Supplementary Fig.3: Amino acid homology of E protein


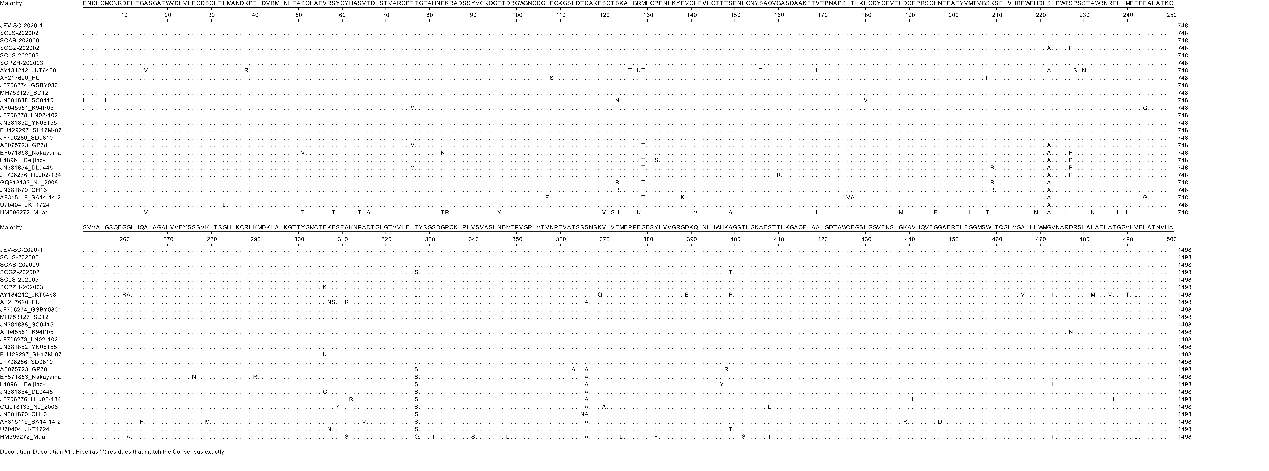


Supplementary Fig.4: Analysis of amino acid mutation of E protein
